# Targeting Sodium Transport Reveals CHP1 Downregulation as a Novel Molecular Feature of Malignant Progression in Clear Cell Renal Cell Carcinoma: Insights from Integrated Multi-Omics Analyses

**DOI:** 10.3390/biom15071019

**Published:** 2025-07-15

**Authors:** Yun Wu, Ri-Ting Zhu, Jia-Ru Chen, Xiao-Min Liu, Guo-Liang Huang, Jin-Cheng Zeng, Hong-Bing Yu, Xin Liu, Cui-Fang Han

**Affiliations:** 1 Guangdong Provincial Key Laboratory of Medical Immunology and Molecular Diagnostics, Guangdong Medical University, Dongguan 523808, China; wuyun@gdmu.edu.cn (Y.W.); zhuriting1998@gdmu.edu.cn (R.-T.Z.); cjrkkl8975@gmail.com (J.-R.C.); liuxiaomin2001@gdmu.edu.cn (X.-M.L.); huangguoliang@gdmu.edu.cn (G.-L.H.); zengjc@gdmu.edu.cn (J.-C.Z.); hbyu@gdmu.edu.cn (H.-B.Y.); 2 School of Pharmacy, Guangdong Medical University, Dongguan 523808, China; 3 Dongguan Key Laboratory of Medical Bioactive Molecular Developmental and Translational Research, Guangdong Medical University, Dongguan 523808, China; 4 Dongguan Key Laboratory of Public Health Laboratory Science, School of Public Health, Guangdong Medical University, Dongguan 523808, China

**Keywords:** clear cell renal cell carcinoma, calcineurin B homologous protein isoform 1, single-cell RNA sequencing, tumor microenvironment, sodium transport, prognostic biomarker

## Abstract

Clear cell renal cell carcinoma (ccRCC), the most common RCC subtype, displays significant intratumoral heterogeneity driven by metabolic reprogramming, which complicates our understanding of disease progression and limits treatment efficacy. This study aimed to construct a comprehensive cellular and transcriptional landscape of ccRCC, with emphasis on gene expression dynamics during malignant progression. An integrated analysis of 90 scRNA-seq samples comprising 534,227 cells revealed a progressive downregulation of sodium ion transport-related genes, particularly CHP1 (calcineurin B homologous protein isoform 1), which is predominantly expressed in epithelial cells. Reduced CHP1 expression was confirmed at both mRNA and protein levels using bulk RNA-seq, CPTAC proteomics, immunohistochemistry, and ccRCC cell lines. Survival analysis showed that high CHP1 expression correlated with improved prognosis. Functional analyses, including pseudotime trajectory, Mfuzz clustering, and cell–cell communication modeling, indicated that CHP1^+^ epithelial cells engage in immune interaction via PPIA–BSG signaling. Transcriptomic profiling and molecular docking suggested that CHP1 modulates amino acid transport through SLC38A1. ZNF460 was identified as a potential transcription factor of CHP1. Virtual screening identified arbutin and imatinib mesylate as candidate CHP1-targeting compounds. These findings establish CHP1 downregulation as a novel molecular feature of ccRCC progression and support its utility as a prognostic biomarker.

## 1. Introduction

Cancer remains a leading cause of premature death and poses a major threat to global public health, with profound social and economic consequences [1]. According to the 2022 GLOBOCAN report, approximately 20 million new cancer cases and 10 million cancer-related deaths were recorded worldwide, with projections indicating a rise to over 35 million new cases annually by 2050 due to population growth and aging. In China, lung, colorectal, thyroid, liver, and gastric cancers are the most prevalent, whereas kidney cancer was ranked as the tenth most commonly diagnosed malignancy among men in 2022. Renal cell carcinoma (RCC), originating from renal tubular epithelial cells, accounts for the majority of kidney cancers and is increasing in incidence by 1.5% annually [2,3]. Among its subtypes, ccRCC is the most common, comprising 65–70% of RCC cases, and is characterized by a high degree of aggressiveness and recurrence [4]. Genetically, ccRCC is frequently marked by deletions of chromosome 3p and loss-of-function mutations in the Von Hippel–Lindau (VHL) gene, which occur in over 90% of cases [5].

VHL mutations are early molecular events in ccRCC and result in the stabilization of hypoxia-inducible factors HIF-1α and HIF-1β, creating a pseudohypoxic state that promotes glycolysis and enhances fatty acid and glycogen biosynthesis. This metabolic reprogramming leads to a lactate-rich microenvironment and widespread intracellular accumulation of cholesterol and other lipids within lipid droplets (LDs). These LDs not only store energy but also regulate lipid uptake, membrane biogenesis, and redox balance, all of which facilitate tumor growth. Immune checkpoint inhibitors (ICIs), particularly those targeting programmed death protein 1 (PD-1) and cytotoxic T-lymphocyte-associated antigen-4 (CTLA-4), such as nivolumab and ipilimumab, have improved clinical outcomes and are now first-line treatments for ccRCC [6,7,8]. However, resistance and relapse remain significant challenges.

Calcineurin B homologous proteins (CHPs) are regulatory cofactors of Na^+^/H^+^ exchangers (NHEs), which maintain intracellular pH homeostasis and influence tumor proliferation, invasion, and apoptosis [9]. Among the three CHP isoforms—CHP1, CHP2, and CHP3—CHP1 was first identified as an essential cofactor for sodium/proton exchanger 1 (NHE1) [10]. Encoded on chromosome 15q15.1, CHP1 is ubiquitously expressed and enhances NHE1 activity through direct binding and glycosylation [11]. CHP1 also interacts with glycerol-3-phosphate acyltransferase-4 (GPAT4), facilitating triglyceride synthesis and endoplasmic reticulum function [12]. These roles underscore the importance of CHP1 in energy storage, membrane formation, and possibly tumor biology. Additionally, altered CHP1 expression has been associated with neurological disorders such as autosomal recessive spinal muscular atrophy (SMA) [13]. CHP2 functions as a tumor-associated antigen in several cancers and was initially identified in hepatocellular carcinoma as HCA520 [14]. CHP3, also known as Tescalcin, is primarily expressed in the heart, brain, and kidneys and has been linked to tissue differentiation [15].

scRNA-seq has revolutionized cancer research by enabling high-resolution transcriptomic profiling at the cellular level. scRNA-seq has been used to reveal cellular heterogeneity, uncover novel cell subpopulations, and facilitate the exploration of the tumor microenvironment (TME), cell–cell communication, and lineage differentiation. When integrated with multi-omics technologies, scRNA-seq provides deeper insights into gene regulation, protein expression, metabolic states, and chromatin structure [16]. These capabilities make scRNA-seq a powerful tool for understanding tumorigenesis, identifying biomarkers, and developing personalized therapies.

Several recent studies have applied scRNA-seq to ccRCC. Alchahin et al. identified a malignant cell cluster enriched in SAA1, SAA2, and APOL1, which correlated with poor survival and metastasis, while cell interaction analyses implicated the CXCL9/CXCL10–CXCR3 and CD70–CD27 axes as therapeutic targets [17]. Another study integrating scRNA-seq, single-cell assay for transposase-accessible chromatin sequencing (scATAC-seq), and WES identified subtype-specific regulatory features that promoted invasion and migration [18]. Zvirblyte et al. characterized a novel vascular cell subpopulation associated with the epithelial–mesenchymal transition (EMT) and poor survival, highlighting clinically relevant stromal–immune interactions [19]. Zhang et al. discovered PDGFR-β^+^GPR91^+^ pericytes as a methionine source for cancer stem cells, driving resistance to tyrosine kinase inhibitors (TKIs). Targeting these cells reduced methionine levels, depleted cancer stem cells (CSCs), and enhanced TKI efficacy [20].

Beyond discovery, scRNA-seq has become integral to drug development. It enables cellular-level target identification, supports biomarker discovery and patient stratification, and aids in understanding drug mechanisms and resistance [21]. With advances in multi-omics integration, decreasing costs, and enhanced computational tools, scRNA-seq is expected to play an increasingly central role in personalized medicine.

Although CHP1 has been implicated in multiple physiological processes, its role in cancer, particularly in ccRCC, remains largely unexplored. Therefore, this study aimed to investigate the expression dynamics, functional role, and molecular mechanism of CHP1 in ccRCC using scRNA-seq, offering new insights into both tumor biology and potential therapeutic strategies.

## 2. Materials and Methods

### 2.1. Collection of Single-Cell Transcriptomic Data

Four scRNA-seq datasets were obtained and curated for this study: GSE178481 [17], GSE207493 [18], and GSE242299 [19] from the Gene Expression Omnibus (GEO) (accessed on 28 August 2024), and PRJNA917036 [20] from the Sequence Read Archive (SRA) (accessed on 28 August 2024). These datasets comprised a total of 64 primary ccRCC samples and 26 adjacent normal kidney samples. Additionally, one spatial transcriptomic dataset (GSE175540) derived from ccRCC tissues [22] was retrieved from GEO (accessed on 10 November 2024) for spatial gene expression analysis.

### 2.2. Preprocessing of scRNA-Seq Data

Initial scRNA-seq data processing was conducted using the Scanpy package (v1.10.3) in Python (v3.8.2) [23], following established protocols (https://scanpy.readthedocs.io/en/stable/tutorials/basics/clustering-2017.html, accessed on 6 September 2024). Low-quality cells and genes were excluded based on the following criteria: genes must be expressed in ≥3 cells, cells must express ≥200 genes, and cells with <500 total transcripts or >20% mitochondrial content were removed. To further enhance data quality, Scrublet was used to detect and exclude doublets and red blood cells [24]. Following quality control, total transcript counts were normalized using the normalize_total function and log-transformed with log1p. Highly variable genes (*n* = 2000) were selected using highly_variable_genes for downstream analyses. Dimensionality reduction was performed using PCA (sc.tl.pca, sc.pl.pca), retaining the top 50 principal components (PCs). To correct for batch effects across samples, the harmony_integrate function was applied [25]. Cell–cell similarities were computed with the neighbors function, and clustering was conducted using the Leiden algorithm with a resolution of 0.4. For visualization, UMAP was employed (umap function) [26]. After preprocessing, the integrated dataset comprised 534,227 cells, including 447,929 tumor cells and 86,298 adjacent normal cells.

### 2.3. Cell Subtype Annotation

Cell clusters were annotated based on Leiden clustering results [27] using canonical marker genes referenced from the CellMarker database (v2.0; http://bio-bigdata.hrbmu.edu.cn/CellMarker, accessed on 6 September 2024) [28]. Marker gene expression patterns were visualized using the dotplot function to depict the distribution of cell subtypes in low-dimensional space.

### 2.4. Mfuzz-Based Expression Pattern Clustering and Functional Enrichment Analysis

To explore dynamic transcriptional changes during tumor progression, the expression profiles of epithelial cells were extracted. Genes missing in more than 25% of cells were excluded. The average gene expression across WHO/ISUP tumor grades was calculated and standardized using the standardize function from the datawizard package. Time-series clustering was then performed using the Mfuzz algorithm (v2.66.0) [29], which grouped genes into 10 clusters based on shared expression trends. Enrichment was performed using the enrichGO function from the clusterProfiler package (v4.14.4) [30,31]. The cnetplot and dotplot functions from the enrichplot package were used for visualization.

### 2.5. Chromosomal Copy Number Variation (CNV) Analysis

Chromosomal CNV inference was performed using the inferCNVpy package (v0.6.0). Gene position annotations were sourced from GENCODE (https://www.gencodegenes.org/human/, accessed on 30 September 2024) [32]. Reference cell types were derived from adjacent normal tissues. CNV profiles were generated by comparing the average gene expression in epithelial cells with that of the reference populations, grouped by chromosomal location. The CNV analysis identified 62,235 tumor cells and 42,802 non-malignant epithelial cells. UMAP plots were used to visualize these subpopulations and to map gene expression patterns associated with key biological processes. Heatmaps were generated to illustrate CNV intensity across chromosomal segments and to visualize the expression of CHP1 across different tumor grades.

### 2.6. Public Database Analysis of CHP1 Expression

Transcriptomic and clinical data for ccRCC patients were obtained from The Cancer Genome Atlas − Kidney Renal Clear Cell Carcinoma (TCGA − KIRC) cohort via the TCGA portal (https://portal.gdc.cancer.gov/, accessed on 28 August 2024). CHP1 expression levels were compared between 542 tumor samples and 72 adjacent normal tissues using Student’s *t*-test. After removing duplicates and incomplete records, a refined dataset of 495 tumor samples and 33 normal tissues was used for subsequent analysis. Protein expression of CHP1 was assessed using UALCAN (https://ualcan.path.uab.edu/index.html, accessed on 10 October 2024) [33], which integrates TCGA and clinical proteomic tumor analysis consortium (CPTAC) proteomic datasets. This analysis validated transcript-level findings and offered additional clinical relevance.

### 2.7. Immunohistochemistry

To assess protein-level expression of CHP1, immunohistochemical (IHC) staining was performed on a tissue microarray (ZLKIC1601, Wellbio, Shanghai, China) containing 80 ccRCC samples and 80 matched adjacent normal tissues. The sections were stained using an anti-CHP1 antibody (1:200 dilution; GTX113936, GeneTex, Irvine, CA, USA). Clinical variables, including sex, age, tumor size, grade, and TNM staging, were available for correlation analysis. The study protocol was approved by the Ethics Committee of Shanghai ZuoLi Biotechnology Co., Ltd. (Approval No. LLSM-15-01, Shanghai, China). IHC images were quantified using Visiopharm software (Version 2023 01.2.13695, Visiopharm, Hoersholm, Denmark), and H-scores were computed to reflect staining intensity and the proportion of CHP1-positive cells [34].

### 2.8. Cell Culture and Western Blotting

HEK293T, human renal tubular epithelial cells (HK-2), and ccRCC cell lines (769-P, A498, Caki-1, and 786-O) were cultured under standard conditions. HEK293T, HK-2, and A498 cells were maintained in DMEM; 769-P and 786-O in RPMI-1640; and Caki-1 in McCoy’s 5A medium. Total protein was extracted using a commercial kit (Takara, Otsu, Japan), and concentration was measured using the BCA method. Western blotting was performed using anti-CHP1 (1:1000, GTX113936, GeneTex, Irvine, CA, USA), anti-FLAG (1:2000, 80801-2-RR, Proteintech, Rosemont, IL, USA), anti-SLC38A1 (1:1000, 28632-1-AP, Proteintech, Rosemont, IL, USA), and anti-β-actin (1:2000, T0022, Affinity Biosciences, Cincinnati, OH, USA) antibodies. HEK293T, HK-2, 769-P, A498, and Caki-1 cells were purchased from Fenghui Biotech Co., Ltd. (Hunan, China), and 786-O cells were purchased from Pricella Biotechnology Co., Ltd. (Wuhan, China) with an STR report.

### 2.9. Prognostic Analysis of CHP1 in ccRCC Patients

To assess the prognostic relevance of *CHP1*, patients from the TCGA-KIRC cohort were stratified into high- and low-expression groups based on the median *CHP1* mRNA level (*CHP1*-High: *n* = 248; *CHP1*-Low: *n* = 247). Kaplan–Meier (KM) survival analysis was conducted using the survfit function from the survival package (v3.5-5), with plots generated by using ggsurvplot from the survminer package (v0.4.9). Univariate and multivariate Cox proportional hazards models were constructed using the coxph function (survival v3.5.5), and forest plots displaying hazard ratios (HRs), 95% confidence intervals (CIs), and *p*-values were generated with the forestplot package (v3.1.1). A prognostic nomogram predicting OS probabilities was developed using the nomogram function in the rms package (v6.7.0).

### 2.10. Pseudotime Trajectory Analysis

The Monocle2 package was used to reconstruct differentiation trajectories of malignant cells [35]. Before analysis, expression data were normalized, denoised, and scaled, and low-quality cells and low-variance genes were removed. A Monocle object was created using the newCellDataSet function. Dimensionality reduction was then conducted with reduceDimension, followed by cell ordering using orderCells. The developmental trajectories of tumor cells were visualized with plot_cell_trajectory, illustrating transitions between distinct cellular states.

### 2.11. CNV Burden Analysis

We first applied the inferCNV algorithm (https://github.com/broadinstitute/inferCNV, accessed on 13 June 2025) to establish a reference set comprising B cells, T cells, NK cells, macrophages, and endothelial cells from the tumor microenvironment, all of which lacked large-scale copy number variations (CNVs). Based on this reference, we computed a cell-by-gene CNV matrix to quantify CNV levels across individual cells. Using this matrix as a baseline, we calculated a CNV score for each cell. To investigate the relationship between CNV burden and CHP1 expression, we then performed Pearson correlation analysis between CNV scores and CHP1 expression levels.

### 2.12. Cell–Cell Communication Analysis

Epithelial cells were divided into *CHP1*-negative (*CHP1*^neg^, *n* = 75,182) and *CHP1*-positive (*CHP1*^pos^, *n* = 29,855) populations based on *CHP1* expression. The CellChat package (v2.1.0) was used to infer communication pathways between *CHP1*^pos^/*CHP1*^neg^ epithelial cells and six other cell types. CellChat identifies biologically significant signaling interactions at the cell subtype level, with a focus on ligand–receptor (L–R) pairs [36]. Both incoming and outgoing signals were inferred, and L–R interaction networks were visualized using the netVisual_heatmap and netVisual_bubble functions.

### 2.13. Spatial Co-Localization Analysis

To estimate the spatial distribution of cell types, deconvolution analysis was performed on the spatial transcriptomic dataset GSE175540 [22] using the Tangram package (v0.4.0) [37]. Tangram integrates scRNA-seq data to predict cell-type proportions and assign scores to each spatial spot based on transcriptomic similarity. Gene–gene expression correlations were further assessed in spatial coordinates. To visualize gene expression density, smoothed spatial distributions were generated using the KernelDensity function from scikit-learn (v1.6.1) [38]. Pearson correlation analysis was applied to evaluate the spatial co-expression of gene pairs. For each sample, smoothed spatial expression values were used to compute correlation coefficients (r) and corresponding *p*-values using the pearsonr function from the scipy package (v1.14.1) [39]. This analysis identified spatially co-expressed or functionally associated gene pairs within the tissue microenvironment.

### 2.14. Differential Gene Expression Analysis

*CHP1* expression was used to stratify tumor cells into *CHP1*^neg^ (*n* = 46,442) and *CHP1*^pos^ (*n* = 15,793) groups. Differentially expressed genes (DEGs) between the two groups were identified using the FindMarkers function in Seurat (v5.0.1) [40], with a threshold of *p* < 0.05 and a log fold change (logFC) > 0. The identified DEGs were subjected to gene set enrichment analysis (GSEA) to explore associated biological pathways.

### 2.15. Protein–Protein Interaction Analysis

To investigate potential interactions between CHP1 and amino acid transporters (AATs), 59 AATs were selected as target proteins [41]. Preliminary screening of protein–protein interaction predictions using AlphaFold3 was performed, with ipTM thresholds greater than 0.6 and predicted template modeling score (pTM) greater than 0.5 (https://alphafoldserver.com/, accessed on 15 December 2024) [42]. Three-dimensional (3D) crystal structures of CHP1 and SLC38A1 were retrieved from the UniProt database (https://www.uniprot.org/, accessed on 26 December 2024) [43]. Protein–protein docking was performed using the HDOCK server (http://hdock.phys.hust.edu.cn/, accessed on 26 December 2024) [44], a hybrid algorithm combining template-based and ab initio strategies. Docking results were visualized using PyMOL [45]. Docking strength was assessed using the docking scores and confidence scores, with lower docking scores and confidence scores > 0.7 indicating stronger binding potential. The model with the lowest predicted binding energy was selected for further analysis. Non-covalent interaction types between CHP1 and SLC38A1 were evaluated using the PLIP platform [46].

### 2.16. Plasmids and Cell Transfection

The coding sequences (CDSs) of human *CHP1* were cloned into expression plasmid pEnCMV-Flag vector, which was purchased from Miaoling Bio (Wuhan, China). The pEnCMV-Flag-*CHP1* fusion expression plasmid was successfully obtained. HEK293T cells were purchased from the Fenghui Biotech Co., Ltd. (Hunan, China) and cultured under standard conditions. Cells were transfected with indicated plasmids using Lipofectamine 3000 (Invitrogen, Carlsbad, CA, USA) according to the manufacturer’s protocol.

### 2.17. Co-Immunoprecipitation Analysis

Forty-eight hours after being transfected with the indicated constructs, HEK293T cells were extracted by TNE buffer (10 mm Tris, pH 8.0, 150 mm NaCl, 1 mm EDTA, 1% NP-40, 10% glycerol with protease inhibitors). Lysates were clarified by centrifugation at 14,000× *g* for 15 min at 4 °C. After centrifugation, the soluble supernatants were incubated with anti-FLAG magnetic beads (Thermo Fisher Scientific, Waltham, MA, USA) overnight at 4 °C. The beads were then washed three times with TNE buffer, eluted by boiling in sample buffer for SDS-PAGE, and at last, subjected to immunoblotting to analyze the indicated antibodies.

### 2.18. Transcription Factor Prediction

Potential transcription factor binding sites in the target gene promoter were predicted using JASPAR (https://jaspar.elixir.no/, accessed on 12 June 2025) with default parameters [47].

### 2.19. Dual-Luciferase Reporter Gene Assay

A dual-luciferase reporter gene assay was performed to investigate the effect of ZNF460 on the promoter activity of CHP1. The luciferase reporter plasmids containing the CHP1 promoter region wild-type (CHP1-WT) and deletion mutant (deletion sequence: GCCTCAGCCTCCCGAG, CHP1-MUT) sequences were constructed and synthesized by Miaoling Bio (Wuhan, China). HEK293T cells were co-transfected with these plasmids, and the luciferase assays were performed after transfection for 48 h by using a Dual-Luciferase Reporter Gene Assay Kit (Promega, Madison, WI, USA).

### 2.20. Virtual Screening and Molecular Docking of FDA-Approved Drugs

Virtual screening was performed to identify potential FDA-approved compounds targeting CHP1. The crystal structure of CHP1 (PDB ID: 7X2U) was retrieved from the RCSB Protein Data Bank (https://www.rcsb.org/, accessed on 20 December 2024) and processed using the “Protein Preparation Wizard” in Schrödinger Maestro (v12.8). The preparation steps included retention of chain C, hydrogen addition, and energy minimization using the OPLS2005 force field (RMSD = 0.30 Å).

Binding pocket prediction was performed using the “sitemap” module, defining a grid box (20 Å × 20 Å × 20 Å) centered on Site1 (GLN77/ALA69/ASP64/HIE116). A total of 3400 compounds were downloaded from the FDA drug database (https://www.accessdata.fda.gov/scripts/cder/daf/, accessed on 20 December 2024), processed via the “LigPrep” module to generate optimized 3D conformations, and screened using the “Glide” module [48]. High-throughput virtual screening (HTVS) was followed by standard precision (SP) and extra precision (XP) docking. The top 20% of compounds at each stage advanced to the next. The docking results were visualized using PyMOL (v2.5).

### 2.21. Molecular Dynamics Simulation

Molecular dynamics (MD) simulations were performed using GROMACS 2022 (version 2022.6, the source code of which is available at Zenodo (URL: https://zenodo.org/records/8134375, accessed on 11 May 2025). Force field parameters were obtained using the pdb2gmx tool and the AutoFF web server. The receptor protein was parameterized using the CHARMM36 force field, while ligand parameters were generated with the CGenFF force field [49]. The system was solvated in a TIP3P cubic water box with a 1 nm buffer in all directions and neutralized by adding counterions via the gmx genion tool [50]. Long-range electrostatic interactions were treated using the Particle Mesh Ewald (PME) method with a 1.0 nm cutoff. All covalent bonds were constrained using the SHAKE algorithm, and integration was carried out using the Verlet leapfrog algorithm with a 1 fs time step. Key structural and dynamic properties were analyzed throughout the simulation. These included root-mean-square deviation (RMSD), root-mean-square fluctuation (RMSF), hydrogen bonding, radius of gyration (Rg), and solvent-accessible surface area (SASA).

### 2.22. Statistical Analysis

Statistical analyses were conducted using Python (v3.8.2), R (v4.3.3), and their associated packages. For normally distributed data, results were presented as mean ± standard deviation and compared using Student’s *t*-test. For non-normally distributed data, medians and interquartile ranges were reported and compared using the Wilcoxon rank-sum test. One-way ANOVA was used to compare means among multiple groups.

Survival analyses were conducted using KM curves, with significance evaluated via the log-rank test. H-score comparisons were performed using GraphPad Prism (v10.12). Pearson and Spearman correlation coefficients were computed using pearsonr and spearmanr from the scipy package (v1.14.1), respectively. All tests were two-sided, with *p* < 0.05 considered statistically significant.

## 3. Results

### 3.1. Cellular Atlas of ccRCC

**Figure 1:**
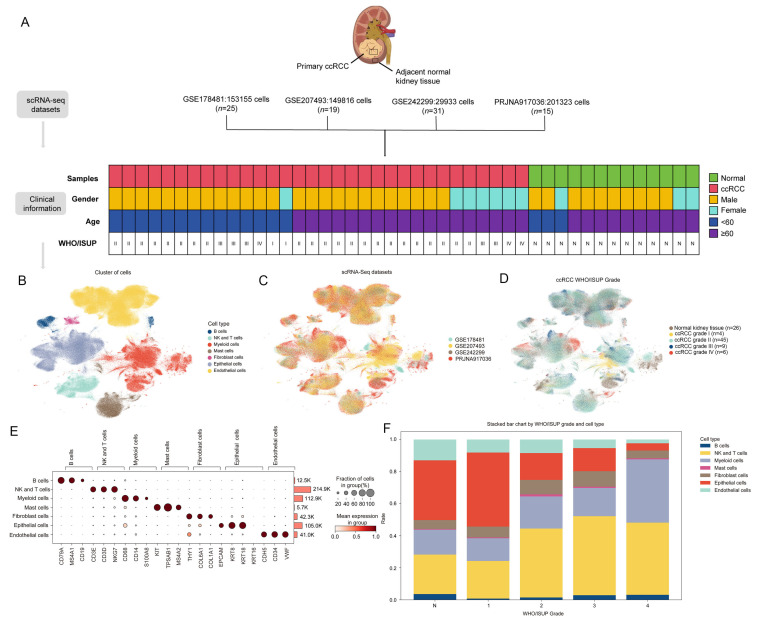
scRNA-seq data collection and identification of cellular subpopulations. (**A**) Overview of the clinical metadata from four integrated scRNA-seq datasets. The flowchart summarizes the number of cells and samples in each dataset, as well as combined statistics including gender, age, and WHO/ISUP grade distribution. (**B**) UMAP projection of 534,227 cells from 64 primary tumor and 26 adjacent normal kidney tissue samples. Each dot represents one cell, and different colors indicate distinct cell subpopulations. (**C**) UMAP plot showing the origin of cells based on the source dataset (GSE178481, GSE207493, GSE242299, or PRJNA917036). Each dataset is represented by a unique color. (**D**) UMAP plot illustrating the distribution of cells by tumor WHO/ISUP grade, including both adjacent normal and primary tumor tissues. Each cell is colored according to its associated grade. (**E**) Dot plot displaying the expression patterns of the top three marker genes for each of the seven cell types. The cell counts per type are shown on the right. (**F**) Bar plot summarizing the relative abundance of each cell type across WHO/ISUP grades. Each bar is segmented by color to represent different cell subpopulations.

To construct a comprehensive single-cell landscape of ccRCC, we integrated four scRNA-seq datasets: GSE178481 (153,155 cells; *n* = 25), GSE207493 (149,816 cells; *n* = 19), GSE242299 (29,933 cells; *n* = 31), and PRJNA917036 (201,323 cells; *n* = 15), comprising 64 primary tumor samples and 26 matched adjacent normal kidney tissues (Figure 1A). In total, 534,227 cells were analyzed, including 447,929 tumor cells and 86,298 adjacent normal cells. Based on canonical marker genes, we identified seven major cell types: B cells (*CD79A*, *MS4A1*, *CD19*), endothelial cells (*CDH5*, *CD34*, *VWF*), epithelial cells (*EPCAM*, *KRT8*, *KRT18*, *KRT16*), fibroblasts (*THY1*, *COL6A1*, *COL1A1*), mast cells (*KIT*, *TPSAB1*, *MS4A2*), myeloid cells (*CD68*, *CD14*, *S100A8*), and NK and T cells (*CD3E*, *CD3D*, *NKG7*) (Figure 1B).

The distribution of the datasets and tumor grades was visualized using UMAP (Figure 1C,D). The dot plots display the expression of representative marker genes across cell subtypes (Figure 1E), and the bar plots illustrate the relative abundance of each cell type across WHO/ISUP grades (Figure 1F). Notably, the proportion of epithelial cells decreased progressively with increasing tumor grade, suggesting a potential link between epithelial depletion and malignant progression.

### 3.2. Gene Expression Profiling of Epithelial Subpopulations During ccRCC Progression

**Figure 2:**
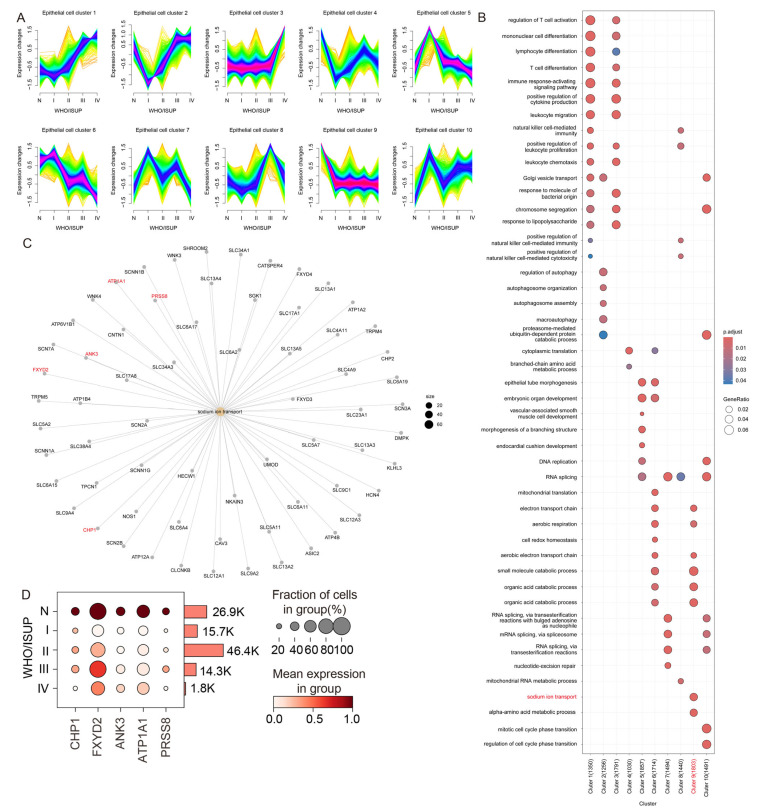
Gene expression profiling of epithelial subpopulations during malignant progression in ccRCC. (**A**) Mfuzz clustering of epithelial cell gene expression across WHO/ISUP tumor grades reveals ten distinct expression patterns. Each curve represents the temporal trend of an individual gene. (**B**) GO enrichment analysis of gene clusters identified by Mfuzz. (**C**) Network diagram of genes involved in the “sodium ion transport” pathway. Red-labeled genes are expressed in more than 10% of epithelial cells, highlighting them as potential key regulators. (**D**) Dot plot showing the expression levels of five representative sodium ion transport-related genes (*CHP1*, *FXYD2*, *ANK3*, *ATP1A1*, and *PRSS8*) across WHO/ISUP grades. The dot color indicates average gene expression, and the cell counts for each grade are summarized on the right.

To analyze gene expression changes during the malignant progression of ccRCC, epithelial cells were selected for further investigation. Based on the gene expression patterns across WHO/ISUP grades, these cells were stratified using the Mfuzz algorithm, which identified ten distinct clusters (Figure 2A). Among them, Clusters 1 and 3 were characterized by increased expression during tumor progression, whereas Clusters 6 and 9 displayed downregulated gene expression.

To understand the biological functions associated with each cluster, GO enrichment analysis was performed. Interestingly, genes involved in sodium ion transport were significantly enriched only in Cluster 9 (Figure 2B). Considering the kidney’s essential role in electrolyte balance and acid–base regulation, this pathway was prioritized for further analysis. A pathway–gene interaction network revealed sixty-four genes associated with sodium ion transport, of which five—*CHP1*, *FXYD2*, *ANK3*, *ATP1A1*, and *PRSS8*—were expressed in over 10% of epithelial cells (Figure 2C). All five genes were found to be significantly downregulated in ccRCC tissues (Figure 2D).

### 3.3. Sodium Ion Transport-Related Genes Are Significantly Downregulated in Malignant Tumor Cells

**Figure 3:**
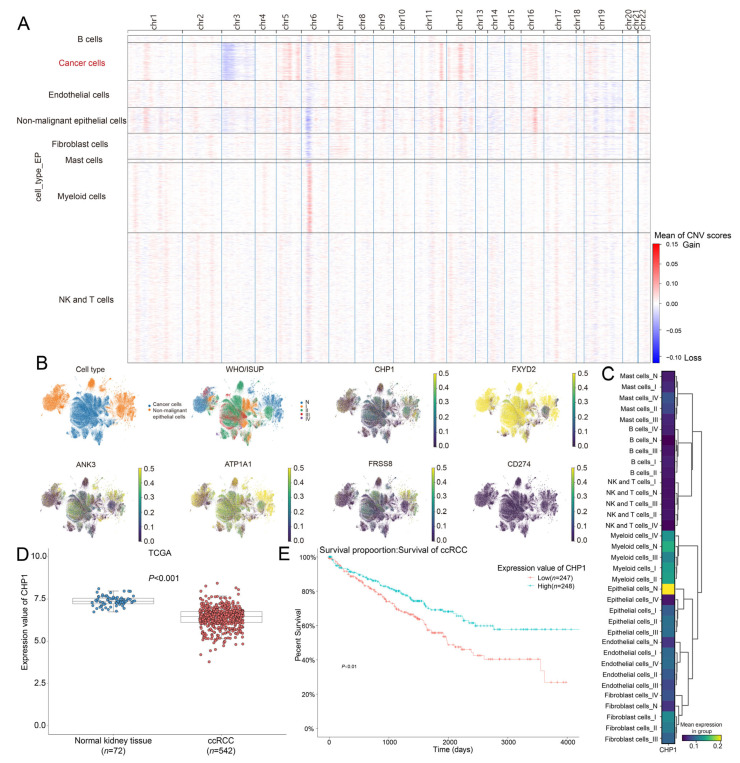
Downregulation of sodium ion transport-related genes in malignant tumor cells and prognostic significance of *CHP1* in ccRCC. (**A**) CNV analysis comparing primary ccRCC and adjacent normal kidney tissues. CNV scores are color-coded by chromosome, with red indicating DNA gains and blue indicating DNA losses. (**B**) UMAP projections displaying the distribution of malignant versus non-malignant epithelial cells, WHO/ISUP tumor grades, and the expression of five sodium ion transport-related genes (*CHP1*, *FXYD2*, *ANK3*, *ATP1A1*, *PRSS8*) and the immune checkpoint marker *CD274*. The gene expression values have been normalized based on transcript count. (**C**) Heatmap of *CHP1* expression across different cell types and tumor grades. The color intensity reflects the average expression level. (**D**) Box plot comparing *CHP1* expression between adjacent normal kidney tissues (*n* = 72) and ccRCC tissues (*n* = 542) in the TCGA-KIRC cohort. Expression values are presented as log2 (TPM + 1); individual dots represent samples. Blue and red dots denote normal and tumor tissues, respectively. (**E**) KM survival curve of OS in the TCGA-KIRC cohort, stratified by *CHP1* expression level. Red and green lines represent *CHP1*-Low and *CHP1*-High groups, respectively.

To distinguish malignant from non-malignant epithelial cells, chromosomal CNV analysis was performed using the remaining seven cell types as reference populations. The CNV heatmaps reveal extensive genomic alterations in malignant cells, including a prominent loss of chromosome 3 and gains in chromosomes 5, 7, and 12 (Figure 3A), which is consistent with known early events in ccRCC pathogenesis; in particular, chromosome 3p deletions were present in over 90% of cases.

A total of 62,235 tumor cells and 42,802 non-malignant epithelial cells were mapped on a UMAP plot (Figure 3B). Their distribution across WHO/ISUP grades confirmed the validity of cell-type classification, with normal tissues primarily aligning with the non-malignant group. We then examined the expression of the five sodium ion transport-related genes in both populations, using *CD274* (PD-L1)—a canonical immune evasion marker—as a comparator. All five genes exhibited marked downregulation in malignant epithelial cells relative to the non-malignant counterparts (Figure 3B).

Focusing on *CHP1*, we observed that its expression was predominantly restricted to epithelial cells and significantly decreased in tumor tissues (Figure 3C). This pattern was validated in the TCGA-KIRC cohort, where *CHP1* expression was significantly reduced in ccRCC samples (*n* = 542) compared with normal tissues (*n* = 72) (Figure 3D, *p* < 0.001). The patients were further stratified into *CHP1*-High (*n* = 248) and *CHP1*-Low (*n* = 247) groups based on the median expression level. KM analysis revealed that higher *CHP1* expression was significantly associated with improved OS (*p* < 0.05) (Figure 3E), suggesting its potential prognostic value in ccRCC.

### 3.4. Reduced CHP1 Protein Expression in ccRCC Tissues and Cell Lines

**Figure 4:**
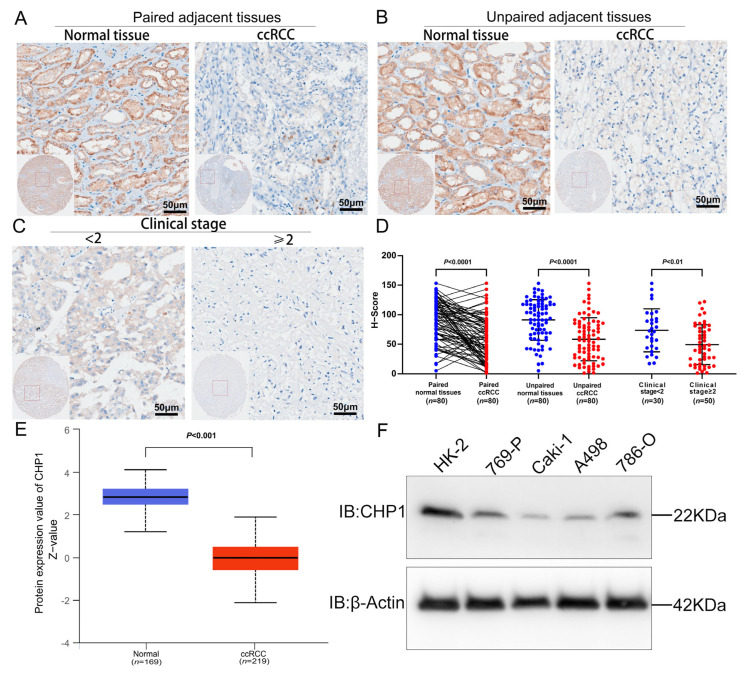
Reduced CHP1 protein expression in ccRCC tissues and cell lines. (**A**) IHC staining showing CHP1 protein expression in matched ccRCC and adjacent normal kidney tissues. (**B**) IHC staining comparing CHP1 protein levels in unmatched ccRCC and adjacent normal tissues. (**C**) IHC results stratified by clinical stage, illustrating CHP1 expression in ccRCC tissues with stages < 2 and ≥2. Scale bar = 50 μm (magnified images). (**D**) Quantitative analysis of IHC H-scores in 80 matched ccRCC and adjacent kidney tissue pairs. Each dot represents an individual sample. (**E**) CHP1 protein expression levels in the CPTAC dataset, comparing between ccRCC and adjacent normal kidney tissues using the Wilcoxon rank-sum test. Blue and red indicate adjacent kidney and ccRCC tissues, respectively. (**F**) Western blot analysis of CHP1 protein levels in 769-P, A498, Caki-1, and 786-O cells relative to HK-2 cells. The original Western blot images can be found in the Supplementary Materials.

To evaluate CHP1 protein expression in ccRCC, we conducted IHC on 80 pairs of tumor and matched adjacent kidney tissues (160 samples in total) and Western blotting on the ccRCC cell lines. The IHC results demonstrate a marked reduction in CHP1 protein levels in ccRCC tissues (Figure 4A,B). Further subgroup analysis revealed that CHP1 expression was lower in advanced-stage samples than in early-stage cases (Figure 4C). Quantitative assessment with H-scores analyzed using Visiopharm software confirmed this trend, showing significantly reduced CHP1 expression in both ccRCC tissues overall and in late-stage tumors (Figure 4D).

Consistent with these findings, data from the CPTAC proteomic database also indicated significantly decreased CHP1 protein levels in ccRCC relative to adjacent kidney tissues (Figure 4E). The Western blot analysis corroborated these observations. Using HK-2 cells as a control, CHP1 protein levels were found to be significantly reduced in all tested ccRCC cell lines (769-P, A498, Caki-1, and 786-O) (Figure 4F).

### 3.5. CHP1 Serves as a Favorable Prognostic Indicator in KIRC

**Figure 5:**
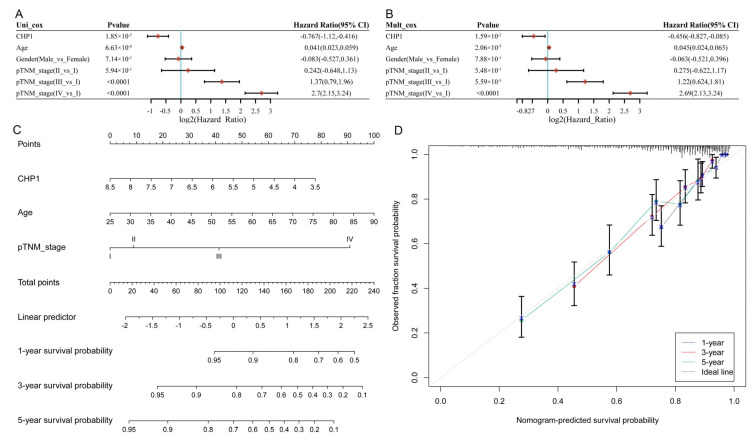
Association between *CHP1* expression, clinicopathological features, and OS in ccRCC. (**A**) Forest plot of univariate Cox regression analysis in the TCGA-KIRC cohort, showing the hazard ratios (HRs) and *p*-values for *CHP1* expression and clinical variables. (**B**) Forest plot of multivariate Cox regression analysis displaying the HRs and *p*-values for the independent variables—*CHP1* expression, age, and TNM stage. (**C**) Prognostic nomogram constructed using *CHP1* expression, age, and pathological TNM stage to estimate the 1-, 3-, and 5-year OS probabilities in ccRCC patients. (**D**) Calibration curves comparing predicted versus observed OS probabilities at 1, 3, and 5 years, demonstrating the predictive accuracy of the nomogram.

To assess the prognostic significance of CHP1 in ccRCC, univariate Cox regression analysis was conducted using data from the TCGA-KIRC cohort (Figure 5A). The results indicate that *CHP1* expression, age, and advanced pathological TNM stage (stage III/IV) were significantly associated with OS, whereas other variables were not statistically significant. Subsequently, multivariate Cox regression analysis confirmed that low *CHP1* expression is an independent prognostic factor, with age and TNM stage III/IV also identified as independent risk factors (Figure 5B).

A prognostic nomogram was constructed incorporating *CHP1* expression, age, and TNM stage to estimate individual risk scores and predict 1-, 3-, and 5-year OS probabilities (Figure 5C). As shown in the figure, low *CHP1* expression, older age, and advanced tumor stage contributed to higher risk scores, which were inversely associated with survival probability. The calibration curves demonstrated the high concordance between predicted and observed OS at 1, 3, and 5 years, supporting the nomogram’s predictive accuracy (Figure 5D).

### 3.6. Gradual Decrease in CHP1 Expression Along the Developmental Trajectory of Tumor Cells

**Figure 6:**
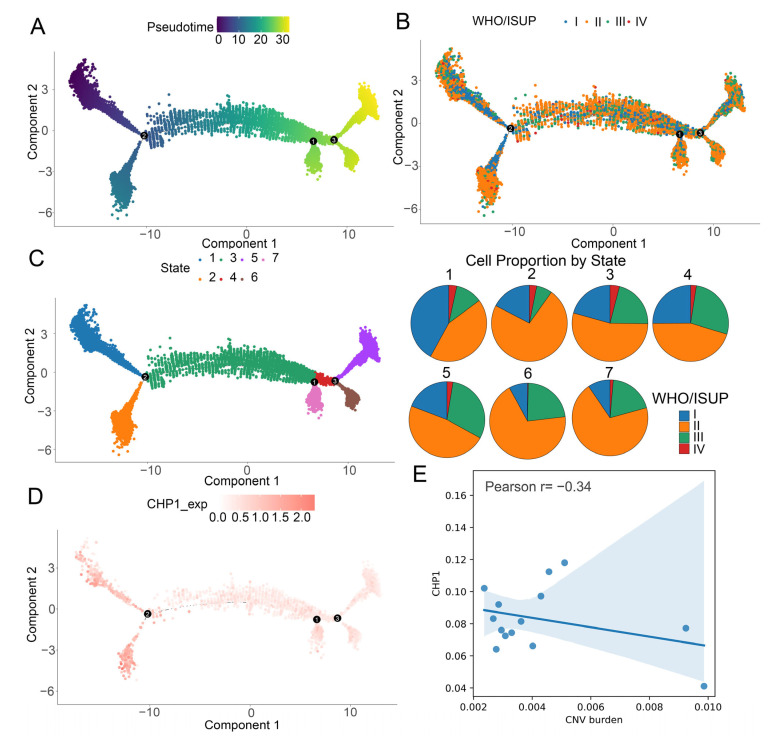
Gradual decrease in *CHP1* expression along the developmental trajectory of tumor cells. (**A**) Pseudotime trajectory of ccRCC tumor cells inferred using Monocle2. Each dot represents a single cell, colored according to pseudotime progression. Numbers in black circles indicate branch points along the trajectory. (**B**) Distribution of WHO/ISUP tumor grades mapped onto the pseudotime trajectory. Each dot represents a single cell, with different colors corresponding to different WHO/ISUP grades. (**C**) Cell group classification along the developmental trajectory. Left: cell stages are colored according to group identity; right: corresponding pie charts displaying the proportion of cells for each WHO/ISUP grade. (**D**) Expression dynamics of *CHP1* along the pseudotime trajectory. Each dot represents a single cell, with the color intensity reflecting the expression level of *CHP1*. (**E**) The relationship between CNV burden and CHP1 expression.

To investigate the temporal dynamics of tumor progression, pseudotime trajectory analysis was performed on ccRCC tumor cells using Monocle2. The inferred trajectory featured three major branch points, reflecting the differentiation continuum of malignant cells (Figure 6A). Tumor cells from WHO/ISUP stage I patients predominantly localized to the early pseudotime regions, while those from stage III and IV patients were enriched in later stages (Figure 6B).

The cells were further categorized into seven groups along the trajectory. The proportion of stage I cells progressively declined, whereas the proportion of stage III cells increased with pseudotime advancement (Figure 6C), consistent with biological tumor progression and validating the trajectory’s relevance.

Notably, *CHP1* expression was predominantly observed in early-stage tumor cells and gradually diminished along the pseudotime axis, indicating a potential role of CHP1 in early-stage tumor biology and differentiation (Figure 6D). In addition, *CHP1* expression was found to be negatively associated with CNV burden (Figure 6E).

### 3.7. Impact of CHP1 Expression on Cell–Cell Communication

To investigate how *CHP1* expression may influence cell–cell signaling in ccRCC, we assessed intercellular interactions by comparing the signaling patterns between *CHP1*-positive (*CHP1*^pos^) and *CHP1*-negative (*CHP1*^neg^) epithelial cells.

**Figure 7:**
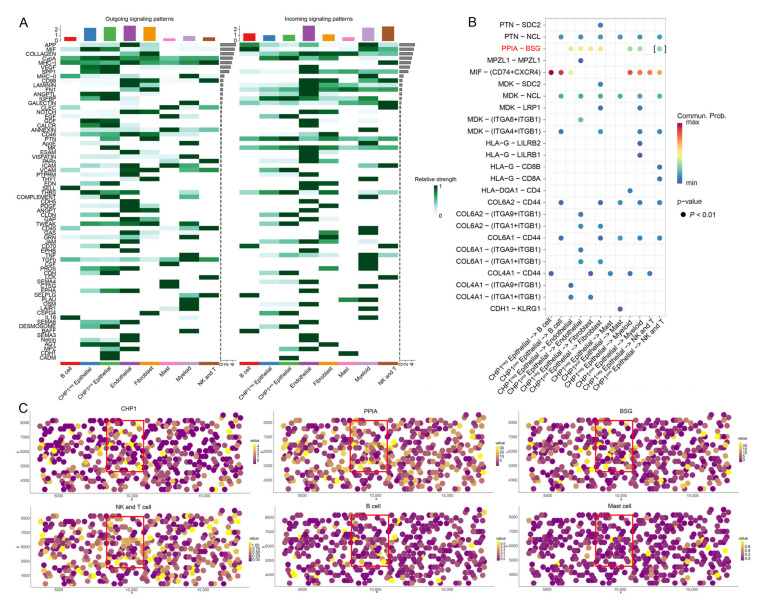
Impact of *CHP1* expression on cell–cell communication in the ccRCC tumor microenvironment. (**A**) Heatmap showing the strength of outgoing and incoming signaling interactions among seven cell populations. Variation in color intensity represents communication probability. The top bar plot summarizes the total incoming and outgoing signal strengths for each cell population, while the right bar plot indicates the overall contribution of each signaling pathway. (**B**) Dot plot depicting ligand-derived signals from *CHP1*^neg^ and *CHP1*^pos^ tumor cells to six other cell types. Dot color represents communication probability, and dot size reflects statistical significance (*p* < 0.01). (**C**) Spatial co-localization analysis using the MISTy framework for three genes (*CHP1*, peptidylprolyl isomerase A *(PPIA)*, basigin *(BSG)*) and three cell populations (NK and T cells, B cells, macrophages). Each dot represents a spatial location within the tissue. Red boxes highlight regions where gene expression and cell populations are co-localized. Axes denote spatial coordinates; gene values indicate expression levels, and cell-type values represent spatial deconvolution scores derived from integrated scRNA-seq data.

Based on single-cell expression data, epithelial cells were stratified into 75,182 *CHP1*^neg^ and 29,855 *CHP1*^pos^ cells. CellChat analysis was applied across all cell types to evaluate outgoing and incoming signaling activities (Figure 7A). *CHP1*^pos^ epithelial cells exhibited enhanced outgoing signals via the MK, MPZ, CDH1, and CADM pathways and stronger incoming signals through the MPZ and CADM pathways compared with *CHP1*^neg^ cells, suggesting greater intercellular communication capacity among *CHP1*^pos^ epithelial cells.

To further discern specific ligand–receptor interactions, we compared *CHP1*^pos^ and *CHP1*^neg^ epithelial cells in terms of their communication with six other cell populations. Dot plot visualization revealed a notably increased interaction between *CHP1*^pos^ epithelial cells and NK and T cells, which was primarily mediated through the PPIA–BSG signaling axis (Figure 7B).

We validated these findings using spatial transcriptomic data to assess the co-localization of *CHP1*, its associated ligand (*PPIA*), receptor (*BSG*), and the relevant cell populations (Figure 7C). The spatial mapping confirmed that *CHP1*, *PPIA*, *BSG*, and NK and T cells were enriched in overlapping regions, whereas B cells and macrophages did not co-localize with *CHP1* expression. These results support the role of PPIA–BSG signaling in mediating interactions between *CHP1*^pos^ epithelial cells and NK and T cells in the ccRCC tumor microenvironment.

### 3.8. CHP1 Expression Affects Amino Acid Transport in Tumor Cells

**Figure 8:**
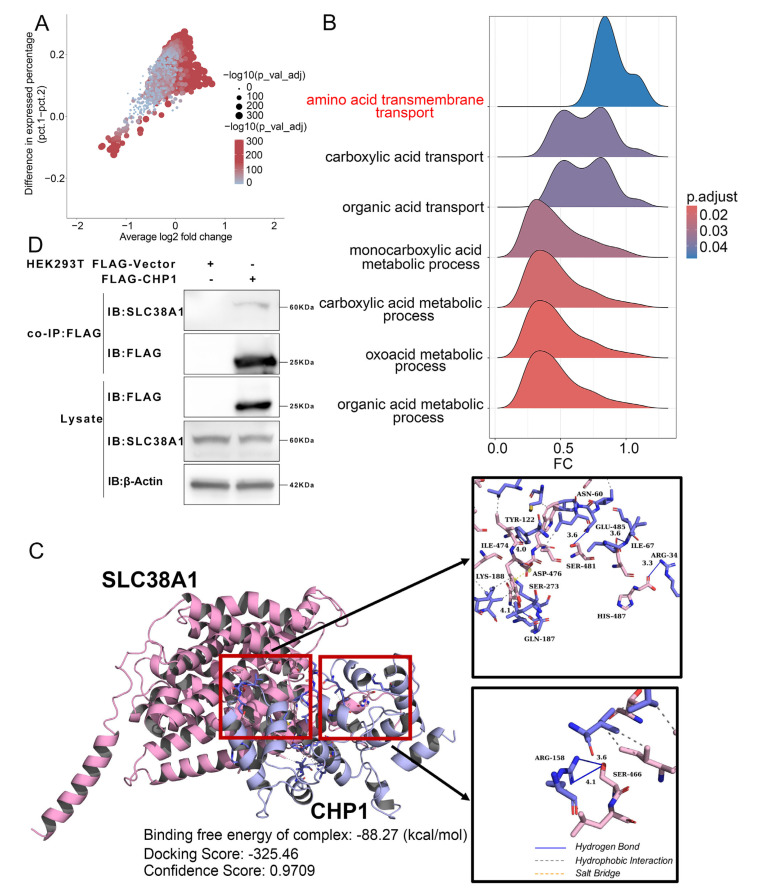
*CHP1* expression influences amino acid transport in tumor cells. (**A**) Differential expression analysis comparing *CHP1*^neg^ and *CHP1*^pos^ tumor cells. Each dot represents an individual gene. (**B**) GSEA of DEGs between *CHP1*^neg^ and *CHP1*^pos^ tumor cells. The colors in the ridge plot correspond to adjusted *p*-values for pathway enrichment. (**C**) Structural visualization of the predicted protein–protein interaction between CHP1 and SLC38A1 using PyMOL. The lower left panel summarizes binding free energy (MM/GBSA), docking score, and confidence score. The right panel shows the predicted non-covalent interactions based on PLIP analysis. Color scheme: SLC38A1 (purple), CHP1 (blue); hydrogen bonds (blue solid lines), hydrophobic interactions (gray dashed lines), and salt bridges (yellow dashed lines). (**D**) CHP1 interacts with SLC38A1. Lysates of HEK-293T cells transfected with FLAG-tagged CHP1 were subjected to immunoprecipitation with anti-FLAG antibody, followed by immunoblotting with anti-FLAG and anti-SLC38A1 antibodies, respectively. The original Western blot images can be found in the Supplementary Materials.

To further elucidate the molecular mechanisms underlying CHP1′s role in ccRCC development, tumor cells were stratified into 46,442 *CHP1*^neg^ and 15,793 *CHP1*^pos^ populations. Differential gene expression analysis using the FindMarkers function identified genes significantly upregulated in *CHP1*^pos^ cells (criteria: *p* < 0.05 and logFC > 0), and subsequent GSEA revealed a strong enrichment of these genes in the “amino acid transmembrane transport” pathway (Figure 8A,B). This result suggests that *CHP1* expression substantially influences amino acid transport processes in tumor cells.

Previous studies have established that *CHP1* acts as a critical auxiliary factor for NHE1—directly binding to its extracellular domain to enhance exchange activity under physiological conditions [11,51]—and that CHP1 interacts with GPAT4, a rate-limiting enzyme in lipid biosynthesis required for ER-based triglyceride synthesis [12]. Building on this multifunctional role, we hypothesized that CHP1 might also affect amino acid transport by interacting with specific amino acid transporters.

To test this hypothesis, we conducted protein–protein molecular docking between CHP1 and 59 known AATs [41]. Among these, CHP1 demonstrated a significant interaction solely with the solute carrier family 38 member 1 (SLC38A1). Using the HDOCK server, docking between CHP1 and SLC38A1 yielded a docking score of −325.46 and a confidence score of 0.9709, indicating a high binding probability. The calculated MM/GBSA binding free energy was −88.27 kcal/mol. Further analysis with the PLIP platform identified seven hydrogen bonds—specifically between SLC38A1-SER466 and CHP1-ARG158, between SLC38A1-HIS487 and CHP1-ARG34, between SLC38A1-GLU485 and CHP1-ILE67, between SLC38A1-SER481 and CHP1-ASN60, between SLC38A1-ILE474 and CHP1-TYR122, and between SLC38A1-SER273 and CHP1-GLN187—and one salt bridge between SLC38A1-ASP476 and CHP1-LYS188 (Figure 8C).

To investigate the interaction between CHP1 and SLC38A1, we performed exogenous co-immunoprecipitation (Co-IP) in HEK293T cells using FLAG-tagged CHP1. The results confirmed that CHP1 binds to SLC38A1 (Figure 8D). These findings suggest that CHP1 may recruit SLC38A1 and regulate amino acid transport.

### 3.9. Prediction of Transcription Factors Involved in Regulating CHP1 Expression

**Figure 9:**
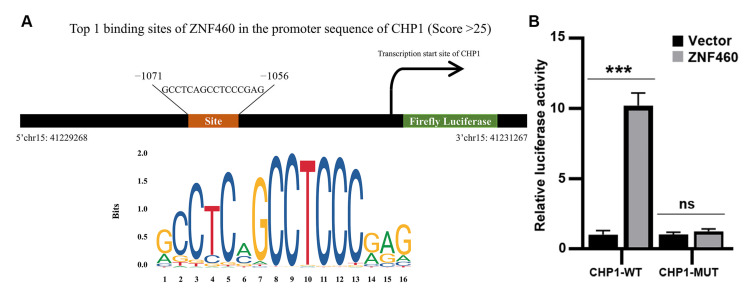
(**A**) The JASPAR database predicted a putative ZNF460 binding site within the CHP1 promoter region. (**B**) Luciferase reporter assay confirmed the direct binding of ZNF460 to the CHP1 promoter. (Data are presented as the mean ± SD, *** *p* < 0.001 and ns, no significance).

Using the JASPAR database, we identified ZNF460 (zinc finger protein 460) as a candidate transcription factor with a relative score > 0.9 (Figure 9A). The binding site with the highest score (−1071 to −1056) was selected for the luciferase reporter assay. The results showed that mutations in the CHP1 promoter sequence significantly reduced transcriptional activity compared with the wild-type sequence (Figure 9B). These findings indicate that ZNF460 acts as a transcription factor that promotes CHP1 expression.

### 3.10. Virtual Drug Screening Identifies Two Compounds Binding to CHP1 Protein

**Figure 10:**
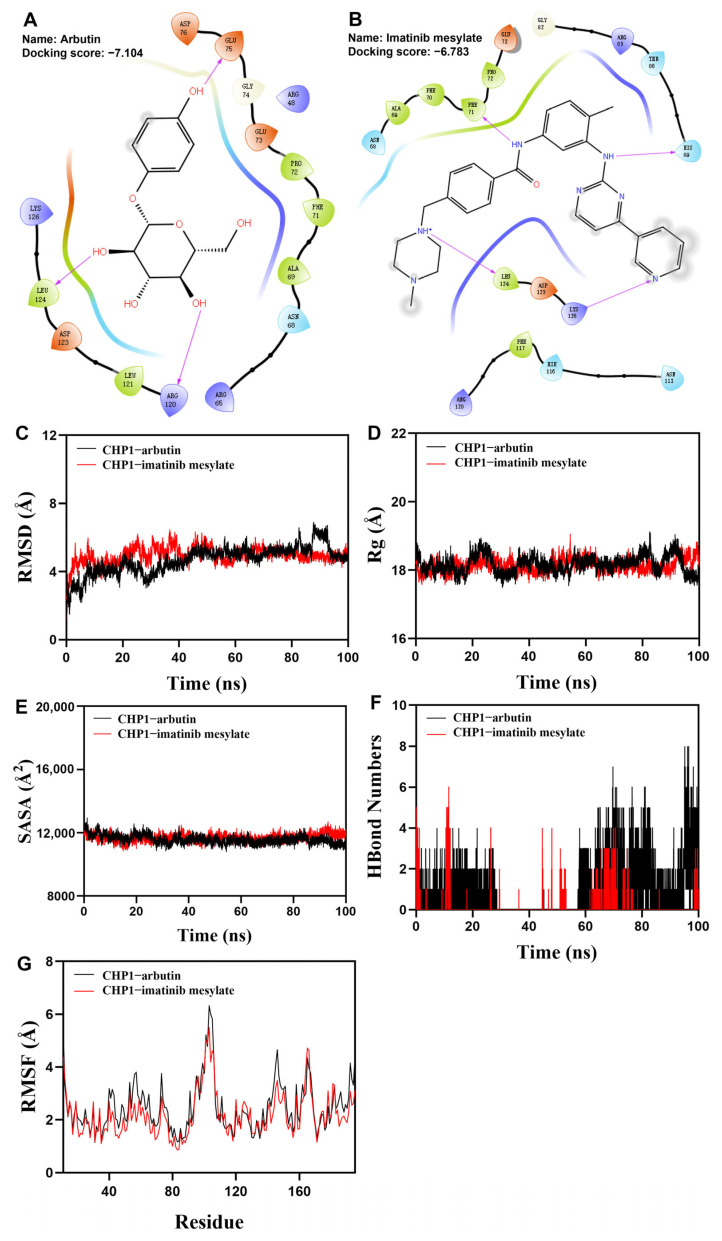
Molecular docking analysis of FDA-approved compounds with the CHP1 protein. (**A**) Predicted binding mode of arbutin with CHP1. (**B**) Predicted binding mode of imatinib mesylate with CHP1. Top left: docking score; center: chemical structure. Color coding: orange = negative charge; purple = positive charge; white = glycine; green = hydrophobic region; blue = polar region. Interaction types: purple solid lines = hydrogen bonds; blue–red solid lines = salt bridges; red lines = π–cation interactions. (**C**) Root-mean-square deviation (RMSD) analysis, (**D**) Radius of gyration (Rg) analysis, (**E**) Solvent-accessible surface area (SASA) analysis, (**F**) Hydrogen bond analysis, (**G**) Root mean square fluctuation (RMSF) analysis of CHP1–arbutin and CHP1–imatinib mesylate.

To identify potential therapeutic compounds targeting CHP1, we performed molecular docking using its crystal structure and an FDA-approved compound library. Docking simulations were carried out in Schrödinger Maestro. Two top-ranking compounds, arbutin and imatinib mesylate, demonstrated high binding affinity to CHP1. Arbutin formed three hydrogen bonds with LEU-124, ARG-120, and GLU-75 on CHP1 (Figure 10A). Imatinib mesylate formed four hydrogen bonds involving LEU-174, PHE-71, and HIS-89 and also exhibited a hydrogen bond interaction between LYS-126 and the nitrogen atom on its pyridine ring (Figure 10B). These results suggest that arbutin and imatinib mesylate may serve as candidate compounds for modulating CHP1-related pathways in ccRCC.

RMSD is a reliable indicator for assessing the conformational stability of proteins and ligands, reflecting the deviation of atomic positions from their initial coordinates. Smaller deviations indicate greater structural stability. Therefore, RMSD was used to evaluate the equilibrium state of the simulation systems. As shown in Figure 10C, the RMSD of the CHP1–arbutin complex stabilized between 45 ns and 90 ns, with a slight upward trend after 90 ns, but overall fluctuated below 4.58 Å. The CHP1–imatinib mesylate complex reached equilibrium after 50 ns and fluctuated around 4.66 Å, indicating that both ligands exhibited stable binding with CHP1. Rg reflects the compactness and global conformational changes in the protein. Larger Rg values typically suggest a more expanded structure. As shown in Figure 10D, both CHP1–arbutin and CHP1–imatinib mesylate complexes exhibited only slight fluctuations in Rg during the simulation, indicating minor conformational changes in the protein–ligand complexes. SASA was calculated to evaluate changes in the protein surface upon ligand binding (Figure 10E). The results showed no significant changes in SASA after ligand binding, suggesting that neither arbutin nor imatinib mesylate induced major structural rearrangements in CHP1. Hydrogen bonds play a crucial role in ligand–protein interactions. As shown in Figure 10F, the number of hydrogen bonds between CHP1 and each ligand fluctuated between zero and six, with an average of approximately one hydrogen bond maintained throughout most of the simulation. This indicates favorable hydrogen bonding interactions between the ligands and CHP1. RMSF was used to evaluate the flexibility of amino acid residues. As shown in Figure 10G, both CHP1–arbutin and CHP1–imatinib mesylate complexes exhibited relatively low RMSF values (mostly below 4 Å), suggesting limited flexibility and high structural stability. In summary, both CHP1–arbutin and CHP1–imatinib mesylate complexes demonstrated stable binding, favorable hydrogen bonding interactions, and minimal structural perturbations. These results indicate strong and stable interactions between arbutin or imatinib mesylate and the target protein CHP1.

## 4. Discussion

Sodium ions play a central role in maintaining osmotic pressure, acid–base balance, and epithelial function. Dysregulation of sodium transport mechanisms has been increasingly linked to cancer development, including ccRCC. The Na^+^/K^+^-ATPase complex, composed of α and β subunits, is essential for ion homeostasis in renal epithelial cells. Both NAKα1 and NAKβ1 are predominantly expressed in the kidney, and decreased NAKβ protein levels have been reported in ccRCC tumor tissues, potentially contributing to tumor aggressiveness [52]. Selvakumar et al. further demonstrated that ATP1B1, the gene encoding NAKβ, undergoes promoter methylation-mediated silencing in RCC cell lines and tissues [53]. Additionally, acid-sensing ion channels (ASICs), a subgroup of the epithelial sodium channel (ENaC) family, are activated under acidic conditions and have been proposed as novel biomarkers and therapeutic targets in ccRCC [54,55]. The FXYD2 gene, which modulates Na^+^/K^+^-ATPase activity, is downregulated in ccRCC and is associated with tumor progression, unfavorable prognosis, and increased regulatory T cells (Tregs) infiltration [56]. Members of the ENaC complex—including SCNN1A, SCNN1B, and SCNN1G—are essential for sodium and fluid homeostasis in epithelial tissues [57]. The expression of SCNN1 genes is reduced at both the mRNA and protein levels in ccRCC, with the expression levels of SCNN1B and SCNN1G being positively correlated with disease progression and poor clinical outcomes [58].

In this study, malignant tumor cells were distinguished from other cell subpopulations based on chromosomal CNV scores, which enabled us to separate them from non-malignant epithelial cells. A comparative analysis revealed that five sodium ion transport-related genes—*FXYD2*, *ANK3*, *ATP1A1*, *PRSS8*, and *CHP1*—were significantly downregulated in malignant cells, implicating their potential involvement in tumor suppression.

FXYD2, located on chromosome 11q23, encodes the γ-subunit of Na^+^/K^+^-ATPase (NAKγ) [59]. It exists in two isoforms—FXYD2a and FXYD2b—and is primarily expressed in the distal tubules of the renal medulla [60]. Interestingly, while FXYD2 is downregulated in ccRCC, it is upregulated in other tumor types, such as colorectal, hepatocellular, and ovarian clear cell carcinomas, where its high expression correlates with advanced disease and poor prognosis [61].

ANK3, a member of the membrane-associated cytoskeletal protein family, functions as an anchoring protein for ion channels and is significantly downregulated in KIRC tissues. Reduced ANK3 expression has been associated with poor survival, supporting its potential role as both a prognostic marker and a modulator of the immune microenvironment [62].

ATP1A1, which encodes the NAKα1 subunit, belongs to the P-type ATPase family. Although it is essential for ionic homeostasis, ATP1A1 is hypomethylated and overexpressed in aggressive tumors such as triple-negative breast cancer (TNBC). Its high expression is associated with poor prognosis in TCGA datasets, and functional studies have shown that ATP1A1 knockout reduces cell viability and tumorsphere formation in TNBC models. These findings suggest a context-dependent role of ATP1A1 across cancer types [63].

PRSS8 encodes a glycosylphosphatidylinositol (GPI)-anchored serine protease that acts as a tumor suppressor. In vivo, conditional knockout of Prss8 in mice leads to spontaneous intestinal inflammation and tumor formation, while PRSS8 overexpression suppresses tumor growth, invasion, and metastasis. Mechanistically, PRSS8 has been shown to inhibit the Wnt/β-catenin, EMT, and cancer stemness pathways, particularly in colorectal cancer [64].

Together, these findings reinforce the notion that sodium transport-related genes, which are downregulated in ccRCC, may play broader roles in tumor suppression, immune regulation, and cancer metabolism. Their altered expression profiles highlight their potential mechanistic links to disease progression and prognosis in ccRCC.

The role of CHP1 in cancer remains largely underexplored. A recent study in hepatocellular carcinoma (HCC) identified a five-gene natural killer cell-related signature (NKRLSig), which included CHP1, and demonstrated that patients in the low-risk group exhibited improved responses to immunotherapy [65]. In our previous work, we developed a glycosylation–tumor microenvironment (GLY/TME) classifier using 15 glycosylation-related genes, where CHP1 emerged as a favorable prognostic marker in HCC. Patients with a low GLY score and a high TME score exhibited better survival outcomes and treatment responses [66].

The pseudotime trajectory analysis revealed that CHP1 is predominantly expressed in early-stage tumor cells, with its expression gradually declining along the developmental timeline, which is consistent with its potential role in tumor suppression. Cell–cell communication analysis showed that *CHP1*^pos^ epithelial cells exhibit more extensive interactions with immune and stromal populations than *CHP1*^neg^ cells. Notably, interaction mapping identified PPIA–BSG signaling as a key communication axis between *CHP1*^pos^ epithelial cells and NK and T cells.

PPIA (peptidylprolyl isomerase A) is implicated in multiple human cancers. Liao et al. reported that PPIA knockdown significantly inhibited invasion and migration in oral squamous cell carcinoma (OSCC) cells, while recombinant PPIA enhanced proliferation and activated the ERK1/2 and p38MAPK pathways through CD147 (BSG) [67]. BSG/CD147 is a multifunctional membrane protein involved in tumor initiation, progression, and metastasis in various cancers, including laryngeal, gastric, renal, and triple-negative breast cancers. Overexpression of CD147 has also been linked to metastatic potential in colorectal, ovarian, cervical, and skin cancers [68]. These findings support the biological plausibility of the CHP1–PPIA–BSG axis as a contributor to immune regulation and tumor progression in ccRCC.

An in-depth analysis of the molecular mechanisms underlying CHP1 function in ccRCC revealed that CHP1 might modulate amino acid transmembrane transport, a process essential to tumor cell survival and metabolism. Protein–protein docking analysis of CHP1 against 59 amino acid transporters identified a specific and high-confidence interaction with SLC38A1, a known glutamine transporter.

Amino acids are not only fundamental to cell structure and metabolism but also critical mediators of cancer progression. AATs maintain intracellular amino acid homeostasis and regulate diverse processes such as pH balance, redox control, energy production, and biosynthesis. Dysregulated AAT activity is a hallmark of metabolic reprogramming in cancer and contributes to tumor growth, immune evasion, and therapeutic resistance [41].

Among AATs, SLC38A1 plays a central role in glutamine transport, and its overexpression is frequently observed in cancers, including breast, lung, colon, and endometrial malignancies [69,70,71,72,73]. In ccRCC specifically, glutamine depletion by tumor cells leads to the activation of HIF1α in infiltrating macrophages, thereby promoting IL-23 secretion and contributing to poor clinical outcomes [74].

The therapeutic potential of targeting CHP1 is further supported by our identification of arbutin and imatinib mesylate as candidate binding agents. Arbutin, a naturally occurring hydroquinone glucoside, is widely recognized for its dermatological applications, but it also exhibits broad-spectrum anticancer, anti-inflammatory, and antioxidant properties, with demonstrated efficacy across various tumor types and minimal toxicity [75]. Imatinib mesylate, a clinically approved tyrosine kinase inhibitor (TKI) for chronic myeloid leukemia and gastrointestinal stromal tumors, is known to interact with transferrin and may be repurposed based on its newly predicted affinity for CHP1 [76,77].

ccRCC remains a therapeutically challenging malignancy due to its high heterogeneity and immunosuppressive tumor microenvironment. Our integrative single-cell analysis identified CHP1 as a previously unrecognized prognosis-associated gene in ccRCC. Given its involvement in sodium homeostasis, pH regulation, lipid metabolism, and amino acid transport, further functional studies on CHP1 may provide new avenues for therapeutic targeting and precision management of ccRCC.

## 5. Conclusions

Our findings highlight CHP1 as a novel prognostic biomarker and functional mediator in ccRCC, with roles in amino acid metabolism, transcriptional regulation, immune cell interaction, and potential drug targeting. These insights warrant further validation and suggest new therapeutic avenues for ccRCC management.

## Data Availability

The data presented in this study are available from the corresponding author upon request.

## Supplementary Materials

The following supporting information can be downloaded at: https://www.mdpi.com/article/10.3390/biom15071019/s1. Title: Original Western Blot Images.

## Abbreviations

scRNA-seq: single-cell RNA sequencing; ccRCC, clear cell renal cell carcinoma; CHP1, calcineurin B homologous protein 1; TCGA, The Cancer Genome Atlas; CNV, copy number variation; CPTAC, clinical proteomic tumor analysis consortium; EMT, epithelial–mesenchymal transition; GEO, Gene Expression Omnibus; GO, Gene Ontology; GPAT4, glycerol-3-phosphate acyltransferase-4; ICIs, immune checkpoint inhibitors; IHC, immunohistochemistry; KIRC, kidney renal clear cell carcinoma; NHE1, sodium/proton exchanger 1; OS, overall survival; TME, tumor microenvironment; AATs, amino acid transporters; TF, transcription factor; Treg, regulatory T-cell; UMAP, uniform manifold approximation and projection; WES, whole-exome sequencing.
