# Supplementary figures and images for: Targeting Sodium Transport Reveals CHP1 Downregulation as a Novel Molecular Feature of Malignant Progression in Clear Cell Renal Cell Carcinoma: Insights from Integrated Multi-Omics Analyses
Source: Biomolecules. 2025 Jul 15;15(7):1019. doi: 10.3390/biom15071019 (PMC12292469; doi:10.3390/biom15071019)

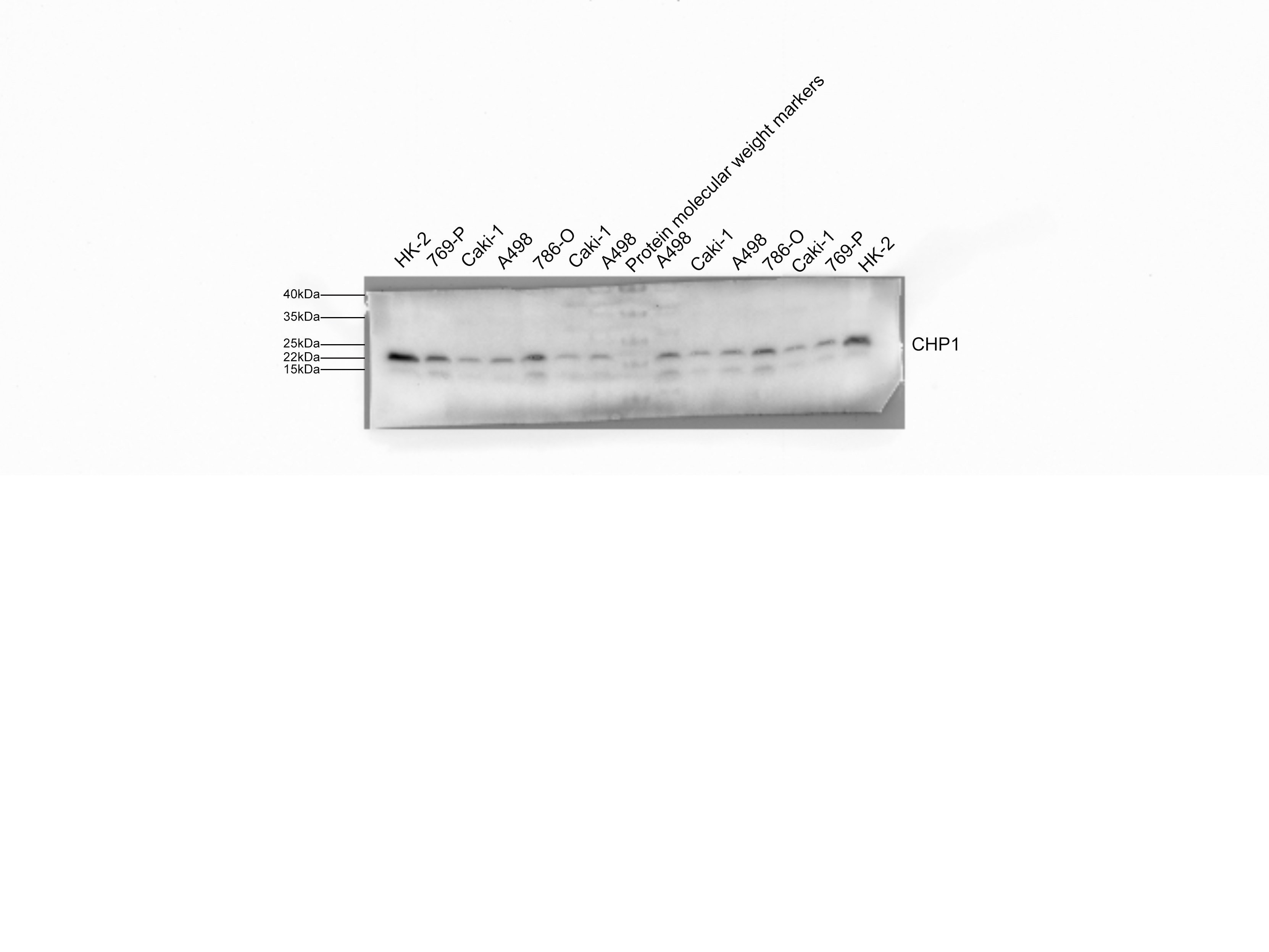

Supplement: Supplementary file 1 [file biomolecules-15-01019-s001.zip › CHP1+marker.tif]

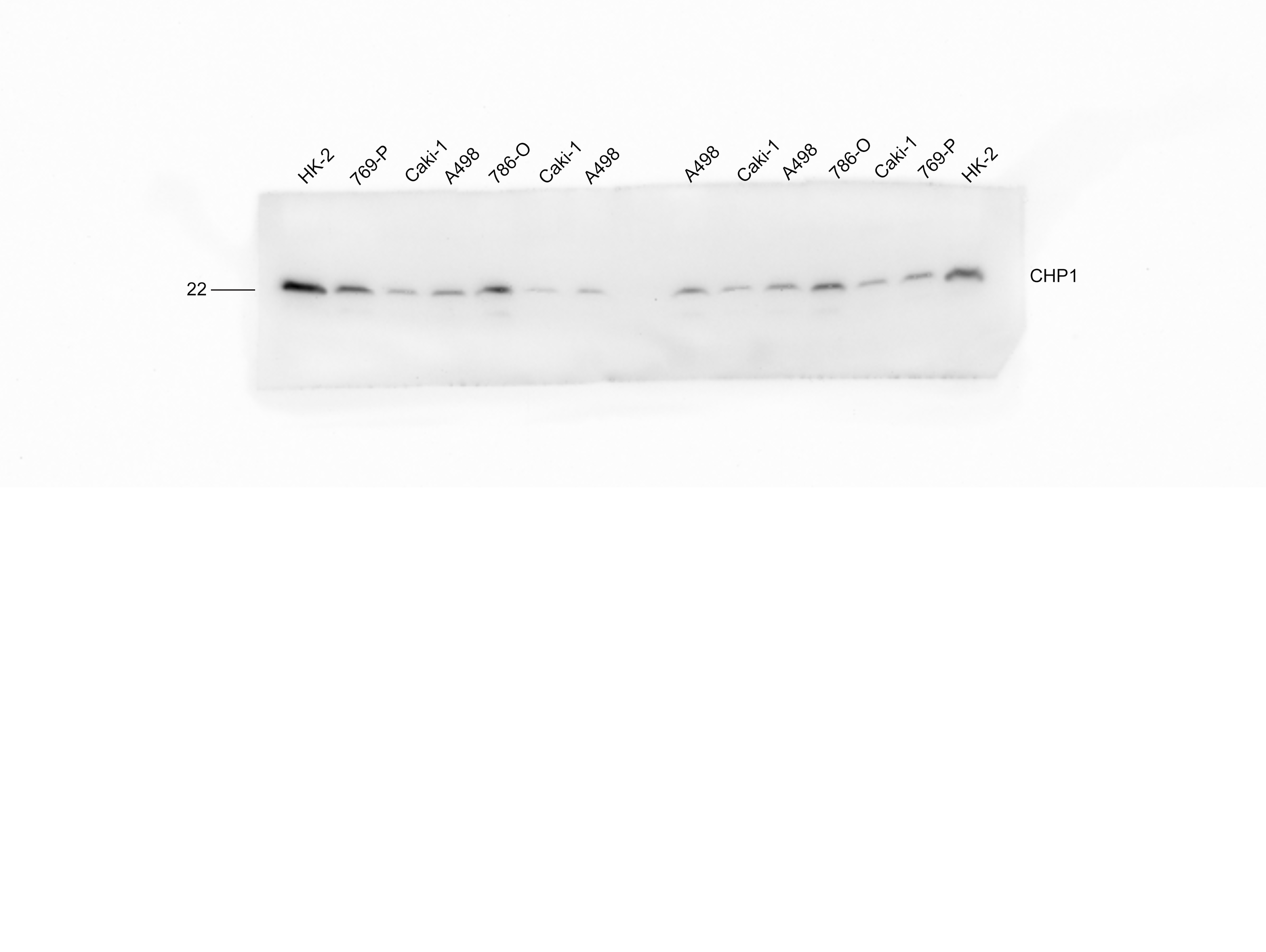

Supplement: Supplementary file 1 [file biomolecules-15-01019-s001.zip › CHP1.tif]

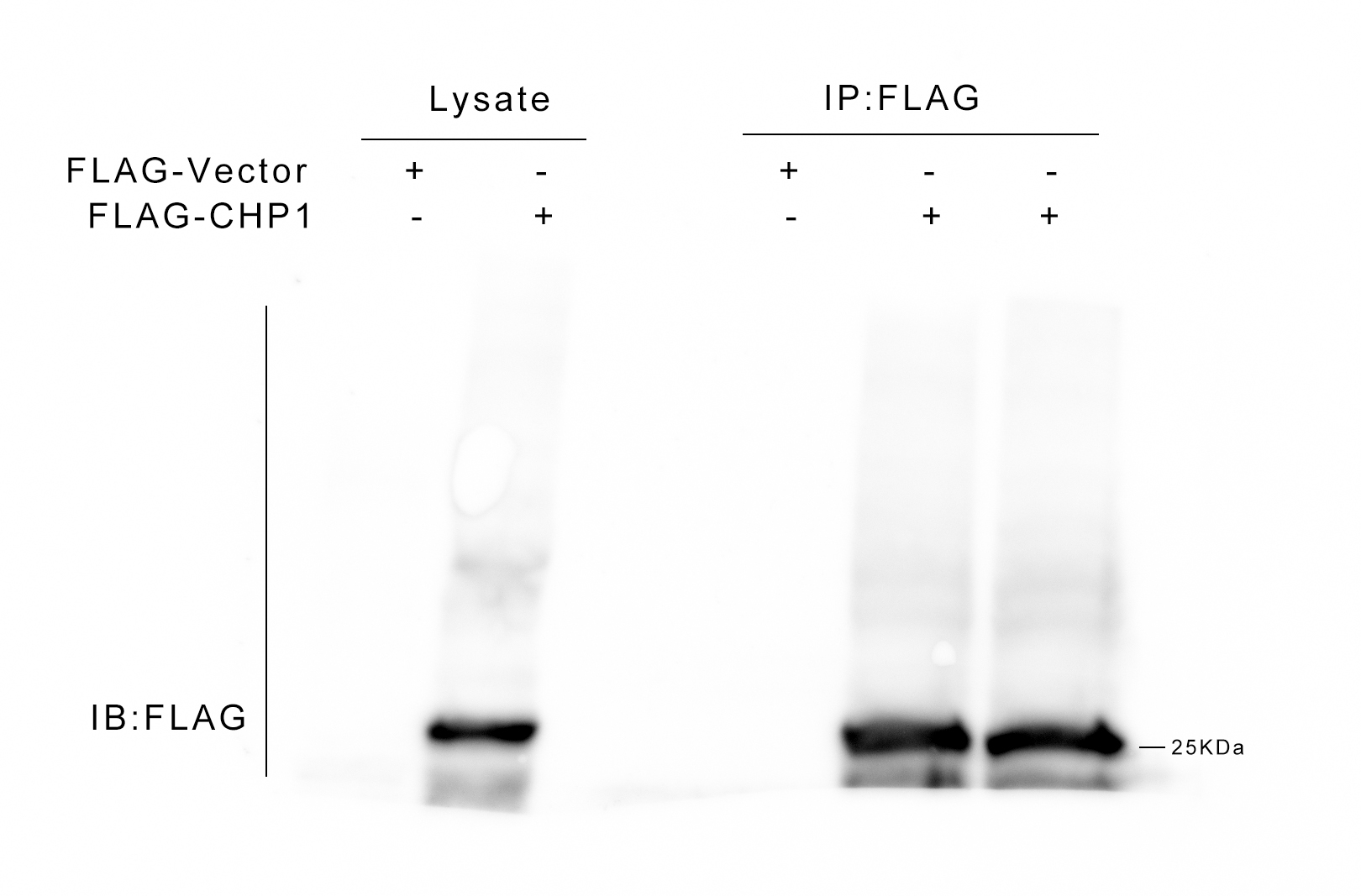

Supplement: Supplementary file 1 [file biomolecules-15-01019-s001.zip › FLAG.jpg]

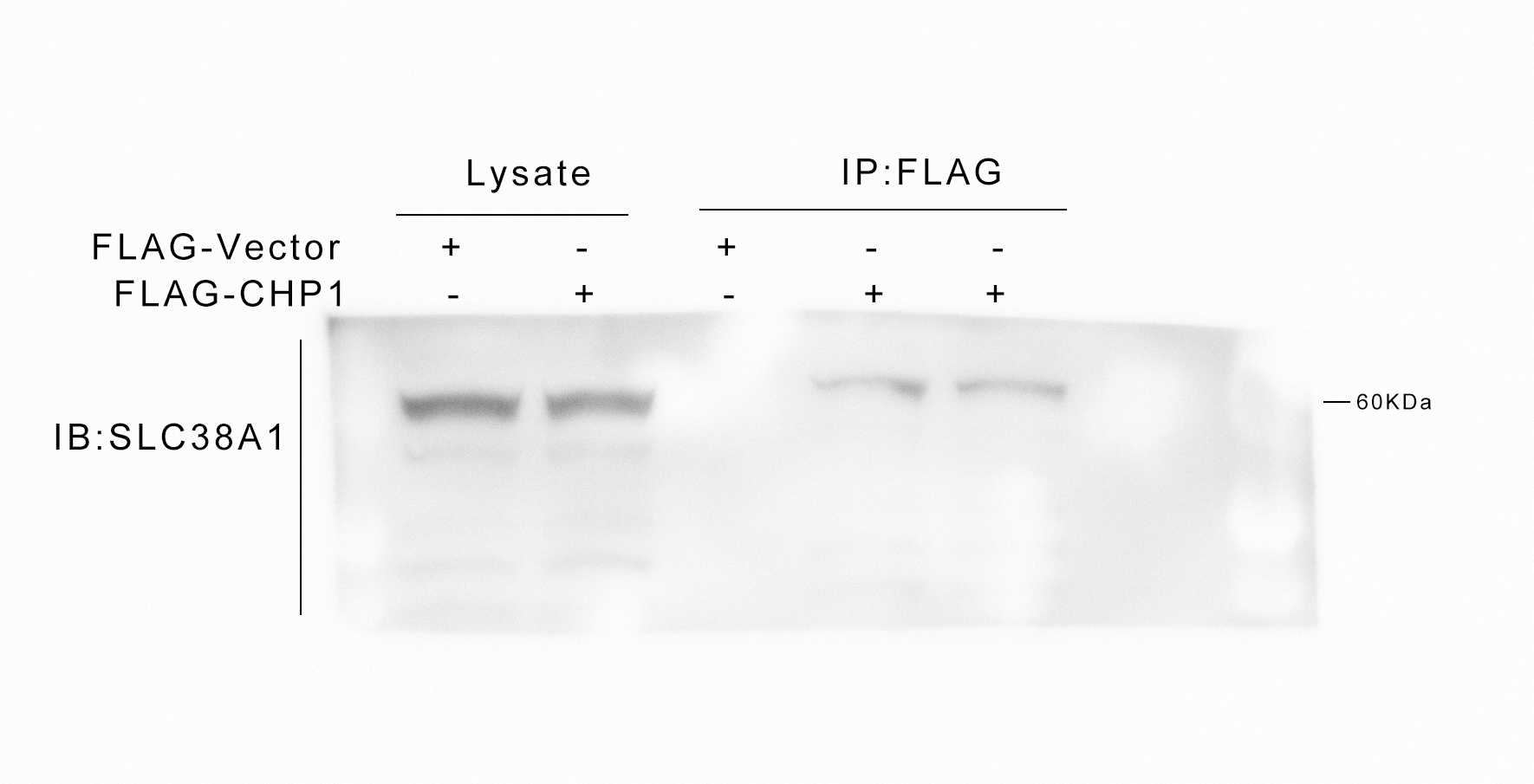

Supplement: Supplementary file 1 [file biomolecules-15-01019-s001.zip › SLC38A1.jpg]

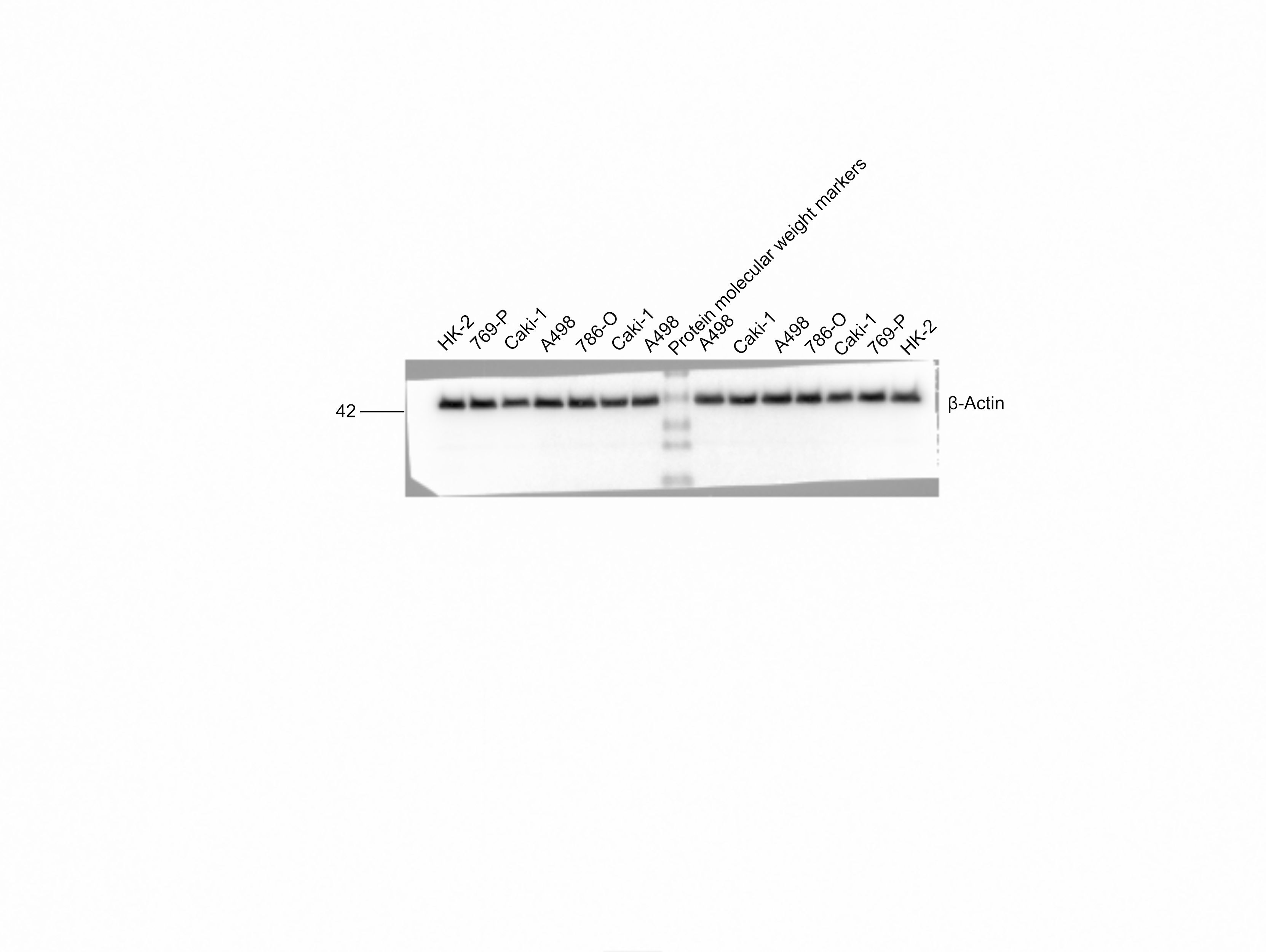

Supplement: Supplementary file 1 [file biomolecules-15-01019-s001.zip › β-Actin+marker.tif]

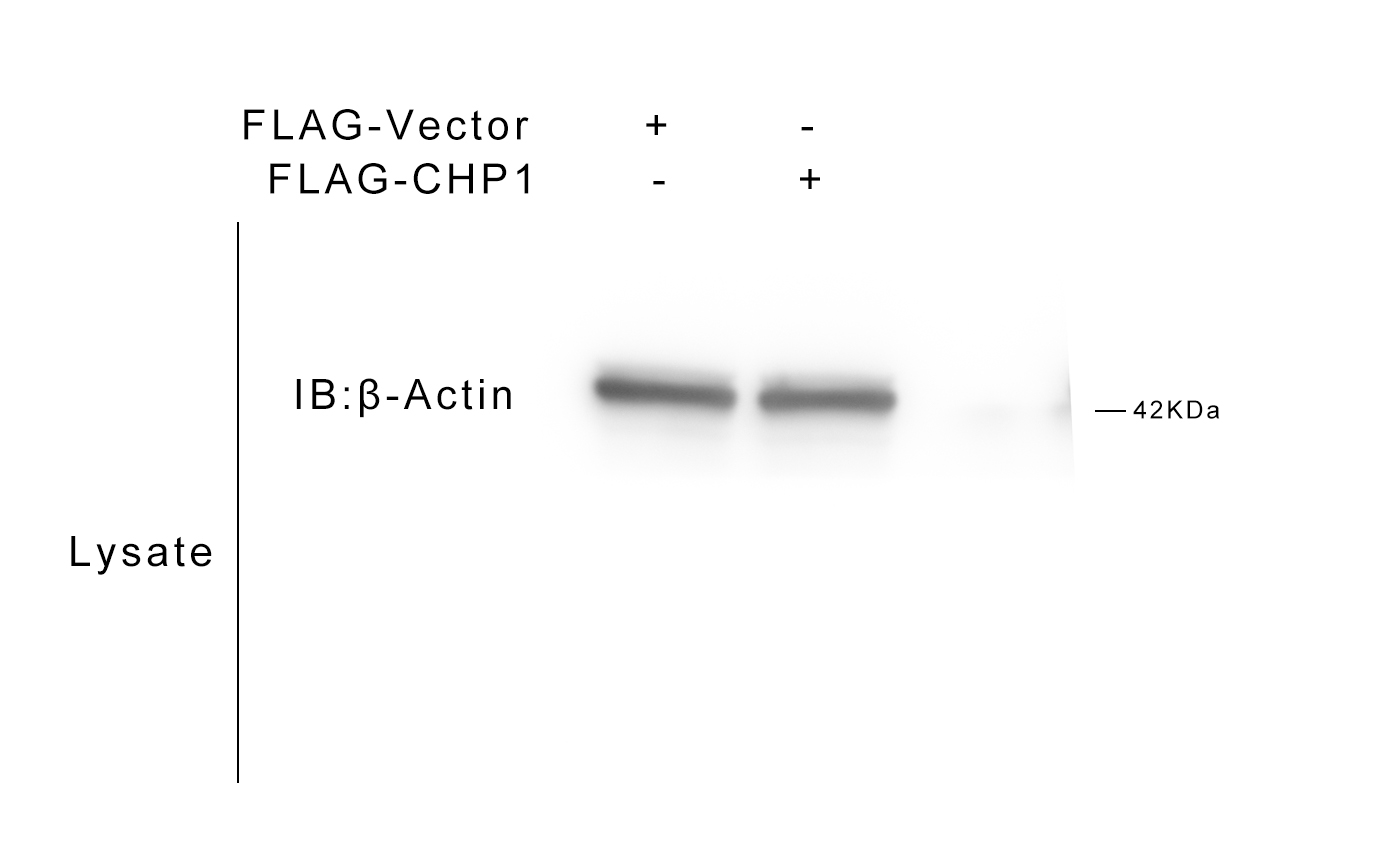

Supplement: Supplementary file 1 [file biomolecules-15-01019-s001.zip › β-Actin.jpg]

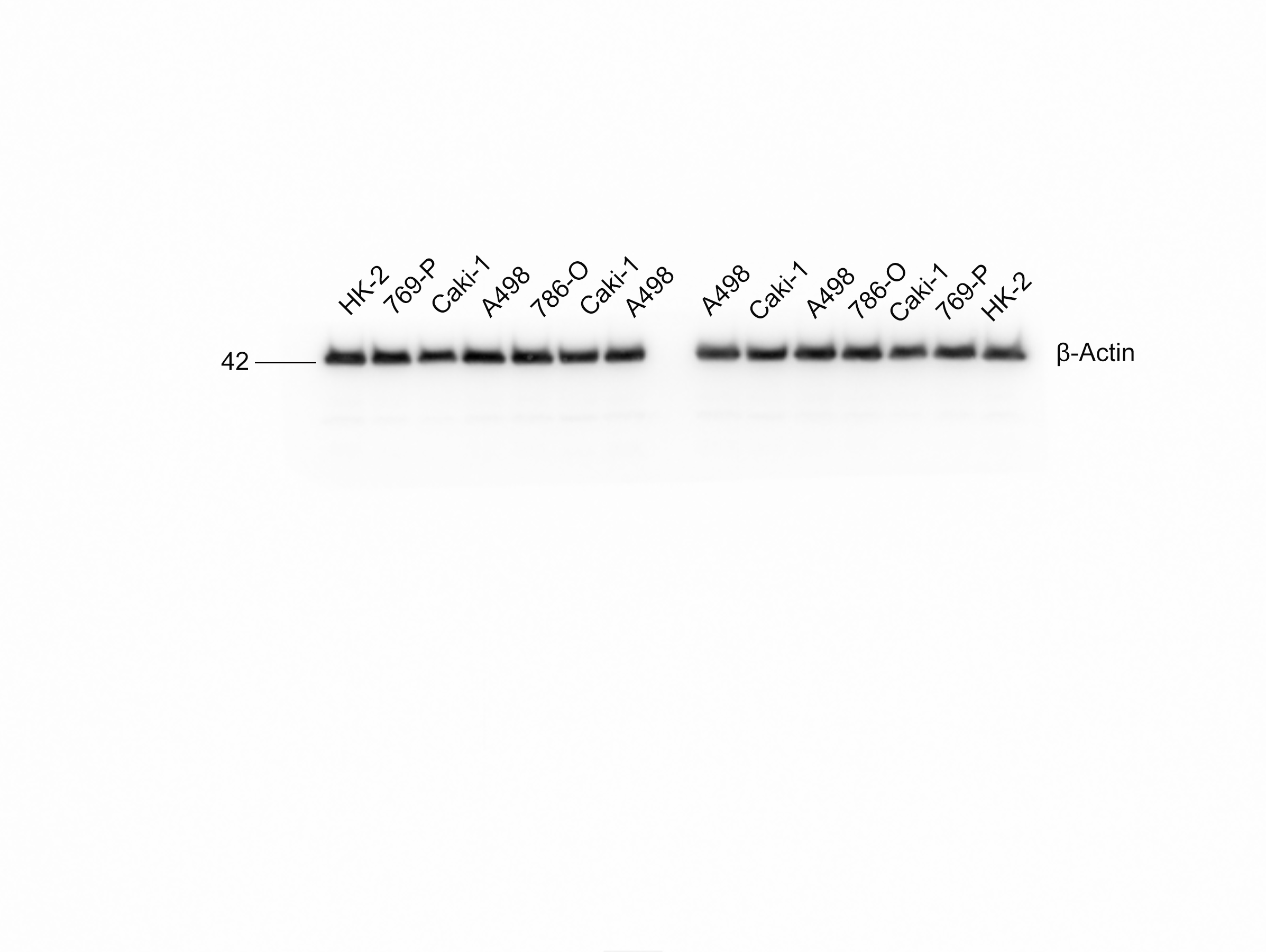

Supplement: Supplementary file 1 [file biomolecules-15-01019-s001.zip › β-Actin.tif]
